# Sugar-Sweetened Beverages and Depressive and Social Anxiety Symptoms Among Children and Adolescents Aged 7–17 Years, Stratified by Body Composition

**DOI:** 10.3389/fnut.2022.888671

**Published:** 2022-05-23

**Authors:** Jieyu Liu, Ting Chen, Manman Chen, Ying Ma, Tao Ma, Di Gao, Yanhui Li, Qi Ma, Li Chen, Xinxin Wang, Yi Zhang, Jun Ma, Yanhui Dong

**Affiliations:** ^1^School of Public Health, Institute of Child and Adolescent Health, Peking University, Beijing, China; ^2^National Health Commission Key Laboratory of Reproductive Health, Beijing, China; ^3^School of Public Health and Management, Ningxia Medical University, Yinchuan, China

**Keywords:** sugar-sweetened beverages, depression, social anxiety, body composition, children and adolescents

## Abstract

**Background:**

Rare studies investigated the associations between sugar-sweetened beverage (SSB) consumption with depressive and social anxiety symptoms among children and adolescents, particularly in different stratification of body composition, which was our purpose.

**Methods:**

A cross-sectional survey of children and adolescents aged 7–17 years was conducted in Beijing, China, in 2020, with an average age of 12.07 (SD: 3.09) years. Children's Depression Inventory (CDI) questionnaires and social anxiety scale for children (SASC) were completed in the baseline questionnaires. SSB consumption and body composition were assessed using child-reported questionnaires and a GE Healthcare Lunar iDXA dual-energy X-ray bone densitometer. Multivariate logistic regression was used to assess the associations between SSB consumption with depressive and social anxiety symptoms. Confounders were evaluated by child-reported and parental questionnaires, including age, sex, parental educational attainment, maternal smoking status, single-child status, BMI, incomes, fruit consumption, physical activity, screen time, and the frequency of fried food consumption. Stratified analyses were performed to explore whether the associations were influenced by body composition.

**Results:**

A total of 1,311 children and adolescents, including 658 boys and 653 girls, were included in the final analysis. There were 13.96 and 29.75% of the study population with depressive and social anxiety symptoms, respectively. Overall, about 63.77% of the children and adolescents consumed SSB, and the average SSB intake was 0.35 servings per day. In the fully adjusted model, compared to participants who did not consume SSB each day, SSB consumption of ≥1 servings/day was positively associated with depressive symptoms [odds ratio (OR) = 2.28, 95% CI = 1.30–4.01] and social anxiety (OR = 1.10, 95% CI = 0.69–1.77), though the latter did not reach statistical significance. When individuals had higher body fat or lower fat-free mass (FFM) or muscle, the ORs of depressive symptoms were more evident among children and adolescents who drank SSB for ≥1 servings/day (*P* < 0.05).

**Conclusion:**

Higher consumption of SSB could be associated with increased OR of depressive symptoms in children and adolescents. The association remained robust, especially in groups with higher body fat or lower fat-free mass or muscle.

## Introduction

Depressive and social anxiety symptoms are the most common mental disorders (1), which are important factors affecting children and adolescents' physical and mental development. During the COVID-19 pandemic, the substantial increase in the prevalence of major depressive and anxiety disorders placed a huge burden on the whole society (2). Depressive symptoms feature low mood, slow thinking, decreased mental activity, cognitive impairment, and physical symptoms (3). Social anxiety is defined as persistent fear or avoidance in social occasions, fear or avoidance arising from comments from others, and fear of embarrassment in social occasions, according to the American Psychological Association (1994) (4). At present, the worldwide prevalence of depression and anxiety is increasing (5), and they are still serious problems for the growth and development of Chinese children and adolescents (6). Notably, several evidence indicated that children with obesity were more likely to suffer from depressive and anxiety symptoms compared to peers of normal weight (7, 8), and fat distribution might be a stronger determinant strengthening such relationship (9). It is, therefore, plausible to speculate that body composition might be correlated with psychiatric disturbances, especially in the case of visceral fat deposits.

Sugar-sweetened beverages (SSBs) has been the major contributor of added sugar in the annual diet, which is detrimental to the health and associated with increased risks of weight gain (10), hypertension (11), and metabolic syndrome (12). In parallel to the physical disorders, SSB consumption might be associated with a modestly higher risk of mental health problems, such as depression among adults (13, 14) and children/adolescent population (15). However, only limited epidemiological studies examined this issue among Chinese children and adolescents. To the best of our knowledge, one study conducted in southern China showed that a higher consumption of SSB was associated with poorer performance on executive function in the pediatric population (16). Meanwhile, a school-based nationwide survey conducted in four provinces of China (i.e., Shenzhen, Zhengzhou, Nanchang, and Guiyang) demonstrated that SSB consumption was associated with depressive symptoms in Chinese adolescents (17). However, they did not consider the confounding effects of lifestyle behaviors, such as diet or physical activities; also, they did not measure adolescent body weight. Overall, one previous report suggested that 66.6% of the Chinese children consumed SSB (18), and the per capita and per consumer SSB intake was 2.84 servings/week (~0.41 servings/day) and 4.26 servings/week (~0.61 servings/day) (18). Since the percentage of consuming SSBs among Chinese children and adolescents is increasing, it is important to consider the potential effect of SSB intake on the risks of mental disorders in Chinese children and adolescents. Furthermore, addressing the influence of potential effect of body composition on the association between SSB consumption and depression/anxiety should also be important when interpreting this topic, when some studies found that body composition seemed to be a better screening tool than relative rough indicators, such as body mass index (BMI) in the prediction of obesity-related diseases (19, 20). Specifically, BMI may overestimate fatness in children who are shorter or who have higher muscle mass and may underestimate adipose in those with reduced muscle mass (21).

Hypothesizing that Chinese children and adolescents who consumed large quantities of SSBs would tend to report more mental health problems, this study aimed to investigate the association between SSB consumption with depressive and social anxiety symptoms among children and adolescents, and further explore the potential effect of multiple body composition indicators on such associations.

## Materials and Methods

### Study Population

We obtained data from a cross-sectional survey conducted among children and adolescents from Beijing, China, in 2020. We adopted a stratified cluster random sampling method, and selected the original study population from elementary school, junior high school, and high school. According to the sample size calculated, a pre-survey was conducted first, and a total of 1,426 children and adolescents aged 7–17 years were invited to participate in the project. For the inclusion and exclusion criteria, individuals with the missing data of SSB intake, body composition, and the definition of depressive and social anxiety were further excluded. A total of 1,311 children and adolescents aged 7–17 years were included in the final analysis. The research of this project was reviewed and approved by the Ethics Committee of Peking University (number: IRB00001052 20024). The school doctor introduced the research purpose and content of the project to all the children and adolescents and their parents in detail, and the written informed consent was obtained from both students and their parents.

### Questionnaire

The questionnaire was distributed by the project team through the school doctors to the students and parents before the physical examination (or through an online questionnaire), with the exception of children in the third grade or under primary school, who completed the questionnaires at home with their primary guardian. Children from or above the fourth grade would fill in the children's questionnaire by themselves while instructed by the class teacher and parents. In addition, trained project members interpreted all the questionnaires in detail. Appropriate guidance would be given by these project members as effectively as possible. The school teacher collected the completed questionnaire on the day of the physical examination and handed it over to our project team members. Child-reported questionnaires included birth date, sex (boys or girls), energy intake behaviors, screen time, and physical activity. Parental questionnaires included information on single-child status (yes or no), parental educational attainment (junior high school or below, high school/secondary or equivalent/junior college or vocational college, undergraduate/postgraduate or above), maternal smoking status (always, quit, and never), and monthly household incomes (<12,000, 12,000–21,000, and ≥21,000 yuan).

For energy intake behaviors, all participants were asked about the frequency (days) and amount of vegetable, fruit, fried food, and SSB consumption. The questions were as follows: “How many days, over the past 7 days, have you drunk SSB, how much have you drunk (serving)?,” “How many days, over the past 7 days, have you eaten fruits/vegetables, how much have you eaten (serving)?,” and “How many days, over the past 7 days, have you eaten fried food?” One serving of vegetable/fruit was defined as the size of an ordinary adult's closed fist and roughly equaled a medium-sized apple (≈200 g) (22). As set in the questionnaire, only the frequency of fried food consumption was recorded, and fried food included fried chicken, deep-fried dough sticks, fried cakes, French fries, and so on. We classified the frequency of fried food consumption into “0–2 days/week,” “3–5 days/week,” and “≥6 days/week.” SSB included Coca-Cola, Sprite, orange juice, Nutrition Express, Red Bull, and all other sorts of sodas and sugary juices. To better understand SSB consumption, one serving of SSB was determined as a canned beverage (~250 ml). The average daily consumption was calculated as follows: (days × servings in each of those days)/7. Therefore, the amount of daily SSB consumption was categorized into groups of “=0 servings/day,” “ <1 servings/day,” and “≥1 servings/day.”

We recorded the child's physical activity according to the International Physical Activity Questionnaire-Short Form (IPAQ-SF) (23) and defined moderate-to-vigorous physical activity (MVPA) as any kind of aerobic activity which increased heart rate and breathing, such as running, swimming, cycling, basketball, football, table tennis, badminton, and calisthenics. According to the questions, “How many days, over the past 7 days, have you done MVPA? And how much time did you last on average?,” children and adolescents were asked to report the frequency (days) and duration (hours and minutes) for MVPA over the past 7 days, and the average daily physical activity was calculated as follows: (days × duration in each of those days)/7.

Screen time was investigated by the following question: “Over the past 7 days, how much time did you spend on watching TV or playing computer or video games on average?,” and students, therefore, reported the daily average duration (hours and minutes) of screen time.

### Depression and Social Anxiety Symptoms

The Children's Depression Inventory (CDI) was a self-report questionnaire consisting of 27 items, including 5 subscales (i.e., Negative Mood, Interpersonal Problem, Ineffectiveness, Anhedonia, and Negative Self-Esteem) (24), which was the most frequently used scale to measure depression in children and adolescents aged 6–17 years (25). The Negative Mood Scale included sadness, worry, self-blame, crying, irritability, and hesitation, corresponding to items 1, 6, 8, 10, 11, and 13; the Interpersonal Problem Scale included bad behaviors, decreased social interaction, disobedience, and quarrels, corresponding to scale items 5, 12, 26, and 27; the Ineffectiveness Scale included self-deprecation, learning difficulties, poor grades, and self-perceived lower than peers, corresponding to scale items 3, 15, 23, and 24; the Anhedonia Scale included unhappiness, sleep disturbance, fatigue, changes in appetite, physical anxiety, loneliness, hating school, and lack of friends, and the corresponding scale items were 4, 16, 17, 18, 19, 20, 21, and 22; the Negative Self-Esteem Scale included pessimism, self-hatred, suicidal ideation, negative body image, and lack of love, corresponding to items 2, 7, 9, 14, and 25. As previously reported, the subscales also had perfect internal consistency (24). The CDI demonstrated good discriminant validity when classifying children and adolescents with no significant psychopathology vs. those who were depressed (24). The items were rated on a 3-point scale indicating symptom severity (0 = no presence of symptom, 1 = symptom is present and mild, and 2 = highest severity possible). The total scores ranged from 0 to 54, and depressive symptoms were defined as CDI scores > 19 (26), with higher scores indicating more severe depressive symptomatology.

The social anxiety scale for children (SASC) (27) was used to assess the social anxiety level of the research subjects, and the scale was applicable to the age of 7–16 years. The scale included 10 items, mainly examining the two dimensions of fear of negative evaluation, social avoidance, and distress. Items 7 and 9 were social avoidance and distress factors, respectively. Each item used a 3-point scoring system, with 0 for never, 1 for sometimes, and 2 for often, with a total score of 20. A total score of ≥8 indicated the possibility of social anxiety, with higher scores indicating a higher degree of social anxiety.

### Physical Examination and Body Composition

Height was measured using a uniform and calibrated mechanical stadiometer (model TZG, Jiangyin No. 2 Medical Equipment Factory, Jiangsu, China), with an accuracy of 0.1 cm. At the same time, the participants were asked to stand straight and barefoot. Weight was measured by a uniform and calibrated electronic scale (model RGT-140, Shanghai Dachuan Electronic Weighing Apparatus Co. Ltd., Shanghai, China) to the nearest 0.1 kg, while subjects were wearing short clothes and standing naturally in the center of the weight measuring plate to keep the body stable. BMI was calculated as the weight (kg) divided by the square of the height (m^2^).

We also measured the children and adolescents' body composition using professional medical personnel using a GE Healthcare Lunar iDXA dual-energy X-ray bone densitometer in accordance with the standard use process and program requirements described by the instrument, scanning the whole body and collecting images. The participants were placed as required, lying flat on the scanning bed, with the body in the middle of the instrument, with the thumb facing up, and the palm facing but not touching the leg. All measurements were logically checked before the examination. During each on-site physical examination, a special person was assigned to conduct on-site supervision to ensure that the measurement methods and records of each measurement index were correct and standardized. Fat mass (FM) was calculated by multiplying weight (kg) by the body fat percentage (BF%). To calculate the fat-free mass index (FFMI), the fat-free mass percentage (FFM%) was calculated by subtracting the BF% from 100%. The fat-free mass (FFM) was calculated by multiplying the weight by the FFM%. The FFMI was then calculated as FFM (kg) divided by height squared, and was useful for comparing individuals with different height measurements (28).

For more precise analysis, we divided age into four groups, namely, 7–9, 10–12, 13–15, and 16–17 years, and then stratified the population into two levels based on the age- and sex-specific median values of body fat mass (BFM), BF%, FFMI, muscle rate, and FFM/FM.

### Statistical Analysis

Continuous and categorical variables were presented as mean ± standard deviation (SD) and frequency (percentage), respectively. Differences in demographic and lifestyle characteristics by sex were examined by Student's *t*-test for continuous variables and Pearson's chi-squared test for categorical variables. Meanwhile, we calculated odds ratios (ORs) [95% confidence level (95% CI)] in two multivariate logistic models to assess the associations between SSB consumption and depressive and social anxiety symptoms. Model 1 adjusted for age and sex; since physical activity and vegetable consumption were collinear variables, Model 2, therefore, additionally included parental educational attainment, maternal smoking status, single-child status, BMI, incomes, fruit consumption, physical activity, screen time, and the frequency of fried food consumption. Furthermore, the cross-sectional survey design could not generate casual relationships of drinking SSBs and depression or social anxiety; therefore, we also exchanged the independent variables with dependent variables to analyze the reverse correlation, in other words, to explore whether individuals with depressive or social anxiety symptoms were more inclined to drink SSB, which could make the results more realistic in terms of causal inference and meaning generalization. Furthermore, stratified analyses were performed to explore whether the associations between SSB consumption and depressive and social anxiety symptoms were influenced by body composition, such as BFM, BF%, FFMI, muscle rate, and FFM/FM. We performed all statistical analyses using the Statistical Analysis System (SAS) software (version 9.4, SAS Institute, Cary, NC, USA), and a two-sided *P* < 0.05 was considered statistically significant.

## Results

### Study Population

Table 1 shows the baseline characteristics of the study population. A total of 658 boys (12.01 ± 3.08 years) and 653 girls (12.13 ± 3.10 years) were included in the final analysis. The mean BMI value was 20.67 (SD: 4.74) and 20.47 (SD: 4.67) kg/m^2^ for boys and girls, respectively. The majority of the study population (65.65%) was single-child status. For the total population, there were approximately 97.56% of children's mothers without smoking, and 42.28% of children's fathers and 43.49% of children's mothers obtained the high levels of education (undergraduate, postgraduate, or above). For lifestyle behaviors, girls (1.26 servings/day) tended to consume more fruits each day than boys (1.17 servings/day, *P* = 0.075), but boys were more likely to take physical activities (0.24 vs. 0.17 h/day, *P* = 0.006) and drink SSB (0.39 vs. 0.31 servings/day, *P* = 0.009), compared to girls. In terms of body composition, girls had more body adipose and high BF%, while boys owned more muscles, and had higher FFMI and a higher ratio of FFM to FM (*P* < 0.0001). Importantly, 13.96 and 29.75% of the study population had depressive and social anxiety symptoms, respectively; overall, separated by sex, girls had higher rates of depressive (16.39%) and social anxiety symptoms (34.92%) than boys (11.55 and 24.62%) with statistically significant difference (*P* < 0.05).

**Table 1:** Baseline characteristics of included population.

**Characteristics**	**Total population (*n* = 1,311)**	**Boys (*n* = 658)**	**Girls (*n* = 653)**	***P*-value**
				
Age, year	12.07 ± 3.09	12.01 ± 3.08	12.13 ± 3.10	0.501
Weight, kg	50.59 ± 18.19	52.64 ± 19.67	48.53 ± 16.32	<0.0001
Height, cm	154.46 ± 15.43	156.90 ± 17.00	152.00 ± 13.23	<0.0001
BMI, kg/m^2^	20.57 ± 4.71	20.67 ± 4.74	20.47 ± 4.67	0.452
Single-child status	858 (65.65%)	448 (68.09%)	414 (63.40%)	0.074
Maternal smoking status, *n* (%)				0.546
Always	20 (1.53%)	8 (1.22%)	12 (1.84%)	
Quit	12 (0.92%)	5 (0.76%)	7 (1.07%)	
Never	1,279 (97.56%)	645 (98.02%)	634 (97.09%)	
Paternal educational attainment, *n* (%)				0.393
Junior high school or below	141 (10.84%)	72 (10.94%)	69 (10.57%)	
High school / secondary or equivalent / junior college or vocational college	620 (47.29%)	299 (45.44%)	321 (49.16%)	
Undergraduate / postgraduate or above	550 (42.28%)	287 (43.62%)	263 (40.28%)	
Maternal educational attainment, *n* (%)				0.099
Junior high school or below	119 (9.16%)	60 (9.12%)	59 (9.04%)	
High school/secondary or equivalent/junior college or vocational college	627 (47.83%)	296 (44.98%)	331 (50.69%)	
Undergraduate/postgraduate or above	565 (43.49%)	302 (45.90%)	263 (40.28%)	
Monthly household income, *n* (%)				0.337
<12,000 yuan	668 (50.95%)	343 (52.13%)	325 (49.77%)	
12,000–21,000 yuan	421 (32.59%)	199 (30.24%)	222 (34.00%)	
≥21,000 yuan	222 (17.18%)	116 (17.63%)	106 (16.23%)	
Lifestyle behaviors				
Fruit consumption, servings /day	1.21 ± 0.93	1.17 ± 0.91	1.26 ± 0.95	0.075
Vegetable consumption, servings /day	1.81 ± 1.28	1.78 ± 1.28	1.85 ± 1.27	0.288
Fried food consumption, days /week	1.26 ± 0.49	1.25 ± 0.47	1.28 ± 0.50	0.297
Physical activity, hours /day	0.21 ± 0.45	0.24 ± 0.48	0.17 ± 0.40	0.006
Screen time, minutes /day	165.44 ± 136.37	165.30 ± 134.00	165.60 ± 138.80	0.971
SSB consumption, servings /day	0.35 ± 0.55	0.39 ± 0.63	0.31 ± 0.46	0.009
Group definition				0.007
0 serving /day, *n* (%)	475 (36.23%)	212 (32.22%)	263 (40.28%)	
<1 serving /day, *n* (%)	719 (54.84%)	379 (57.60%)	340 (52.07%)	
≥1 servings /day, *n* (%)	117 (8.92%)	67 (10.18%)	50 (7.66%)	
Body composition, median (IQR)				
BFM	13.68 (10.52)	12.28 (10.45)	14.87 (10.02)	<0.0001
BF%	29.88 (11.63)	26.04 (13.51)	31.96 (9.35)	<0.0001
FFMI	13.91 (3.70)	14.63 (4.19)	13.35 (2.95)	<0.0001
Muscle rate	0.67 (0.12)	0.71 (0.14)	0.65 (0.09)	<0.0001
FFM/FM	2.34 (1.37)	2.85 (1.93)	2.17 (0.92)	<0.0001
Depressive symptoms, *n* (%)	183 (13.96%)	76 (11.55%)	107 (16.39%)	0.012
Social anxiety, *n* (%)	390 (29.75%)	162 (24.62%)	228 (34.92%)	<0.0001

*BMI, body mass index; SSB, sugar-sweetened beverage; BFM, body fat mass; BF%, body fat percentage; FFMI, free-fat mass index; FFM/FM, fat-free mass/fat mass*.

*One serving of fruit/vegetable roughly equaled 200 g, and one serving of SSB was determined as ~250 ml*.

### Rate of Depressive and Social Anxiety Symptoms by SSB Consumption Stratification

**Figure 1:**
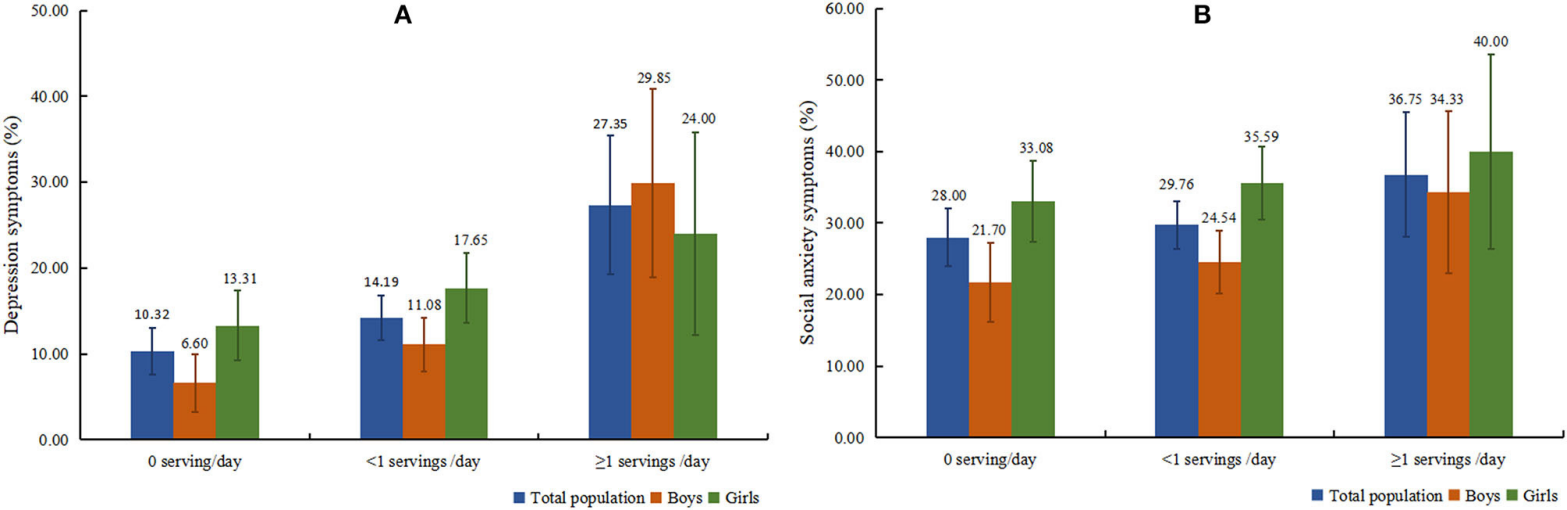
Prevalence of depressive **(A)** and social anxiety **(B)** symptoms among total population and boys and girls, respectively.

The rates of depressive and social anxiety symptoms by SSB consumption stratification are shown in Figure 1. In general, the prevalence of depressive and social anxiety symptoms of individuals with SSB intake ≥ 1 servings/day accounted for a large percentage (depression = 27.35%; social anxiety = 36.75%), followed by individuals with <1 servings/day (depression = 14.19%; social anxiety = 29.76%) and 0 serving/day (depression = 10.32%; social anxiety = 28.00%). Separated by sex, the prevalence of social anxiety symptoms was higher among girls than boys in all groups, while only boys drinking SSB for ≥1 servings/day had a slightly higher prevalence of depression (boys = 29.85%, girls = 24.00%).

### Association Between SSB Consumption With Depressive and Social Anxiety Symptoms

Table 2 presents the ORs and 95% CI of SSB consumption for depressive and social anxiety symptoms. After adjusting for age and sex, compared to the group with SSB consumption of 0 servings/day, the ORs (95% CI) of depressive symptoms in group with SSB consumption of ≥1 servings/day were 2.83 (1.59–5.02) and 4.65 (2.00–10.82) among the total population and boys, respectively. SSB intake of ≥1 servings/day was also positively associated with depressive symptoms in girls but with no statistical difference (OR = 1.63, 95% CI = 0.71–3.73). After additionally adjusting for other covariates in Model 2, the results did not change essentially. In terms of social anxiety, SSB consumption of ≥1 servings/day could also increase the OR of social anxiety but failed to reach statistical significance (Model 1: OR = 1.40, 95% CI = 0.87–2.25; Model 2: OR = 1.10, 95% CI = 0.69–1.77).

**Table 2:** Multivariate odds ratios (ORs) and 95% confidence intervals (CIs) of SSB consumption for depressive and social anxiety symptoms.

**Disorders**	**SSB Consumption**		
	**0 serving/day**	** <1 servings/day**	**≥1 servings/day**
***Model 1***			
**Total population**			
Depressive symptoms	1 (Reference)	1.38 (0.90–2.10)	**2.83 (1.59–5.02)**
Social anxiety	1 (Reference)	1.11 (0.83–1.49)	1.40 (0.87–2.25)
**Boys (*****n*** **=** **658)**			
Depressive symptoms	1 (Reference)	1.42 (0.70–2.88)	**4.65 (2.00–10.82)**
Social anxiety	1 (Reference)	0.97 (0.62–1.51)	1.48 (0.77–2.87)
**Girls (*****n*** **=** **653)**			
Depressive symptoms	1 (Reference)	1.39 (0.82–2.35)	1.63 (0.71–3.73)
Social anxiety	1 (Reference)	1.25 (0.85–1.85)	1.23 (0.62–2.46)
***Model 2***			
**Total population**			
Depressive symptoms	1 (Reference)	1.21 (0.82–1.79)	**2.28 (1.30–4.01)**
Social anxiety	1 (Reference)	0.99 (0.75–1.30)	1.10 (0.69–1.77)
**Boys (*****n*** **=** **658)**			
Depressive symptoms	1 (Reference)	1.32 (0.68–2.57)	**3.42 (1.47–7.92)**
Social anxiety	1 (Reference)	0.95 (0.62–1.46)	1.14 (0.58–2.24)
**Girls (*****n*** **=** **653)**			
Depressive symptoms	1 (Reference)	1.19 (0.72–1.95)	1.57 (0.70–3.52)
Social anxiety	1 (Reference)	0.96 (0.66–1.39)	1.00 (0.51–1.96)

*Model 1: adjusted for age and sex*.

*Model 2: additionally adjusted for parental educational attainment, maternal smoking status, single-child status, BMI, incomes, fruit consumption, physical activity, screen time and the frequency of fried food consumption. Bold values referred to P < 0.05*.

We also reported the reverse correlation that whether individuals with depressive and social anxiety symptoms were more inclined to drink SSB ≥ 1 servings/day, as shown in Supplementary Table 1. Obviously, adjusting for confounders in Model 2, boys with depressive symptoms were more likely to drink SSB for ≥1 servings/day (OR = 2.86, 95% CI = 1.45–5.64), compared with their counterparts without depressive symptoms. In addition, boys with social anxiety also presented more SSBs drinking each day (OR = 1.20, 95% CI = 0.65–2.20), though no statistical significance was observed.

### Distribution of Body Composition

**Figure 2:**
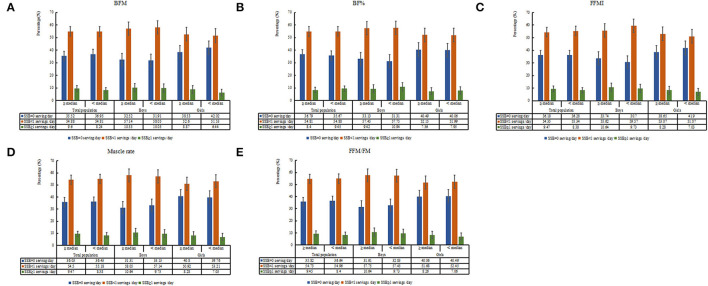
Distribution of sugar-sweetened beverage (SSB) consumption grouped by body composition among total population and boys and girls, respectively (**A**: BFM stratification; **B**: BF% stratification; **C**: FFMI stratification; **D**: Muscle rate stratification; **E**: FFM/FM stratification).

The distribution of body composition among the total population and each sex group is displayed in Figure 2. Based on the age- and sex-specific median value of BFM, BF%, FFMI, muscle rates, and FFM/FM ratios, the total population was divided into two groups almost equally, and the specific percentage of participants in each group was shown in the figure. Overall, in each body composition stratification, ~35–36% of individuals did not drink SSB each day, 54–55% of individuals consumed less than 1 servings/day, and 8–9% of individuals drank equal or over 1 servings/day.

### Potential Effect of Body Composition on the Association Between SSB and Depressive and Social Anxiety Symptoms

**Figure 3:**
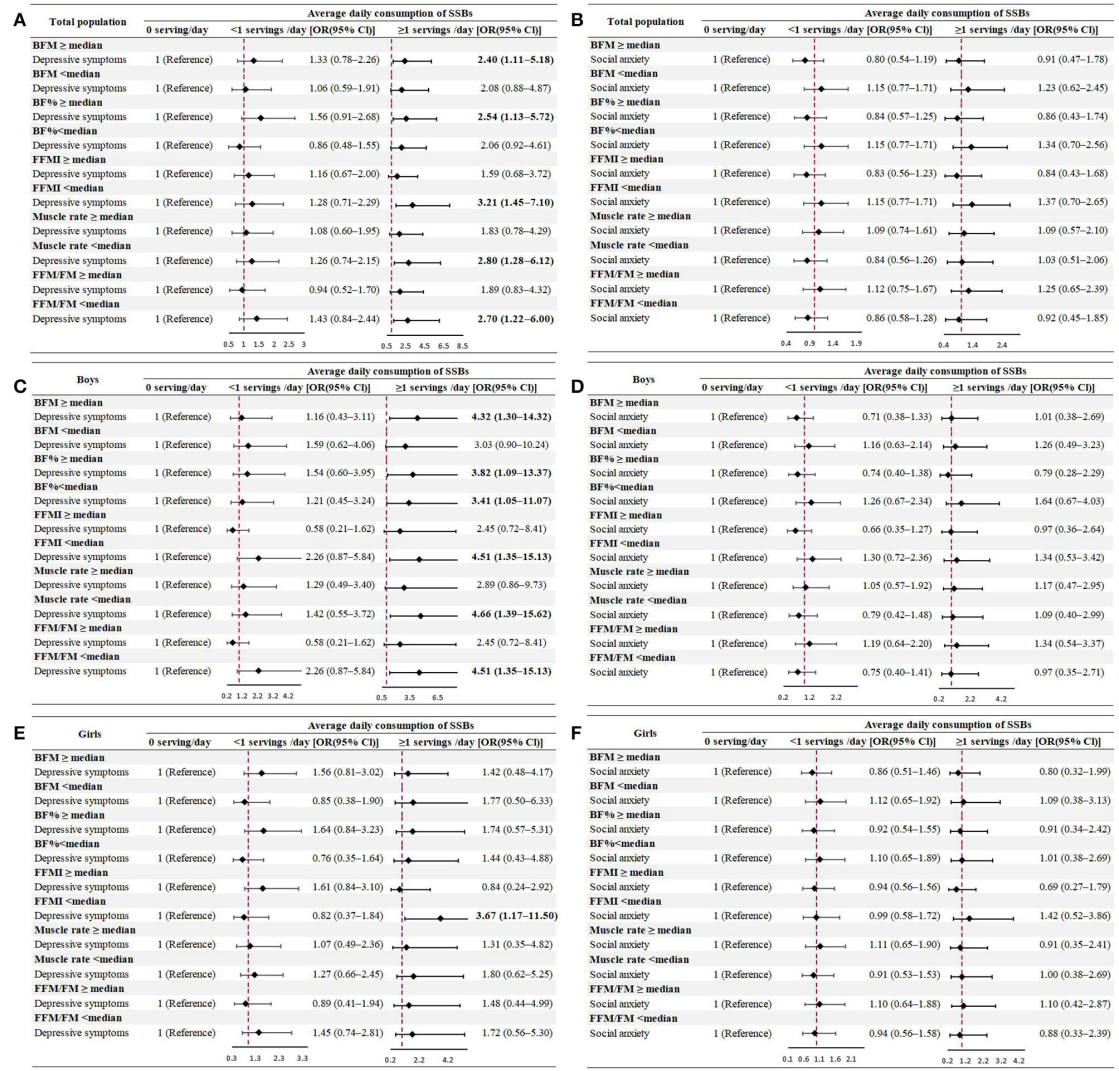
Multivariate odds ratios (OR) and 95% confidence intervals (CI) of SSB consumption for depression (**A,C,E**) and social anxiety (**B,D,F**) symptoms among the total population and each sex group, stratified by body composition (model was adjusted for age, sex, parental educational attainment, maternal smoking status, single-child status, BMI, income, fruit consumption, physical activity, screen time, and the frequency of fried food consumption).

Figure 3 shows the stratified associations between SSB consumption and depressive and social anxiety symptoms by body composition stratification. Obviously, when individuals had higher BFM and BF%, lower FFMI, muscle rates, and FFM/FM ratios, the ORs of depressive symptoms were more evident among the total population and boys who drank SSB for ≥1 servings/day (*P* < 0.05), compared to their peers who did not drink SSB each day.

As for the reverse correlation, similar results were observed. Children and adolescents with mental disorders who had higher body fat or lower FFM or muscle showed a strong tendency to drink SSB for ≥1 servings/day (*P* < 0.05) (Supplementary Figure 1).

## Discussion

In the present cross-sectional survey in Beijing, more than one-half of children and adolescents (63.77%) consumed SSB every day and their average SSB intake was 0.35 servings per day. Meanwhile, such population had 13.96% of depressive symptoms and 29.75% of social anxiety. To the best of our knowledge, higher consumption of SSB was positively associated with increased OR of mental disorders, especially depressive symptoms in male children and adolescents. In addition, children and adolescents with high body fat or low FFM or muscle could be a high-risk population.

In view of SSB consumption and mental health problems, limited evidence existed and yielded inconsistent observations. Prospective studies suggested that frequent consumption of SSB might increase a depression risk among older US adults (14) and Japanese adults (29). One meta-analysis also revealed a non-linear dose-response relationship, and the inflection point was the equivalent of about 2 cups/day of cola (13). However, no significant association between SSB consumption and depression risk was found among Spanish university graduates (30). Among the pediatric population, consumption of SSB was positively associated with depressive symptoms in Chinese adolescents (17), and secondary school children of Dhaka city, Bangladesh (31). On the contrary, based on an autoregressive cross-lagged path model, Mrug et al. found that soft drink consumption at age 13 predicted fewer depressive symptoms among US adolescents (32). These inconsistent findings might be attributed to the different sample size, ethnicity, study design, definition of SSB consumption, and depressive symptoms. In addition, we found positive associations between drinking SSBs and social anxiety, though failing to reach statistical significance. Apart from a large quantity of influencing factors, the limited amount of SSB consumption of the Chinese youth might also be the reason for the non-significant result. There were hardly any studies that investigated the association between SSB and social anxiety; therefore, we could not discuss this issue based on the previous findings.

Interestingly, boys might be more vulnerable to SSB-induced depression, and we speculated several potential explanations for the observed sex differences. First, these results might be related to higher consumption of SSB per day as well as less intake of fruits and vegetables among boys, compared to girls. Second, the results might reflect differences in pathways of depression by sex (33) and type of depressive symptomatology (34). Third, women were more in favor of psychotherapy than men (35), which might weaken the effects of diet behaviors on mental disorders, to some extent. Besides, differences could also be due to the limitations of the cross-sectional design.

Notably, although the results were typically interpreted in terms of SSB contributing to emotional problems, it was equally likely that mental health problems might be driving the consumption of sugar-sweetened soft drinks. Indeed, experimental studies showed that some individuals consumed more sugary foods in response to stress and negative emotions (36). Our study presented that individuals with mental disorders were more likely to drink SSB. However, a previous study has come to the opposite conclusion and has suggested that depressive symptoms did not contribute to a greater frequency of soft drink consumption over time (32). These differences might be attributed to study population (sex, age, ethnicity, etc.), study design, analytical method, and the definition of SSB or mental health problems. Based on the present evidence, interventions, such as limiting SSB intake, could prove to be beneficial for children's and adolescent's mental health; meanwhile, targeted at high-risk population with mental disorders, restricting the consumption of SSB could also prevent the feedback-induced increases or development of mental illness, since they were more likely to drink SSB.

Not surprisingly, more evident associations between SSB consumption and depressive symptoms were observed among individuals with higher body fat or lower FFM or muscle. Certainly, significant relationships between obesity or high fat and depression appeared, and the magnitude of the association remained stronger in children than adults (37). Weight-related deficits in self-esteem as well as stigma and discrimination were often experienced among children and adolescents with obesity (38), which might contribute to the development of depression (39). This lack of attention to mental health and depression caused by unsatisfied body shape is concerning given our findings.

The physiological mechanisms through which SSB might contribute to mental disorders remained to be established. Obviously, SSB contains a large amount of added sugar, which can increase hypothalamus–pituitary–adrenocortical (HPA) axis reactivity leading to elevations in glucocorticoids (40), and drinking high-level SSB has the potential to promote long-term dysregulation of the stress response. Besides, sugar included in SSB might cause higher secretion of proinflammatory cytokines and lead to inflammation (41), while the latter is positively correlated with the incidence of depression (42). Apart from this, SSB consumption is partly responsible for obesity, while obesity might be associated with the development of mental illness through a stimulation of the HPA axis (43). Although speculative, there are some mechanistic reasons to consider that added sugar contained in SSB might directly impact the development of mental health problems. As for the reverse correlation, one possibility is that individuals with mental disorders might crave sweet beverages, and one might speculate that this might occur even years before receiving a diagnosis of mental illness.

Despite known health risks, reducing SSB consumption is still facing some difficulties. Some possible reasons might be related to sugar-induced addiction through consumption of SSB and attractive marketing strategy (44). Our study provided an important implication for public health. First, governments around the world should implement the SSB taxes, since SSB taxes introduced in jurisdictions appeared to have been effective in reducing SSB purchases and intake (45). Importantly, tax-induced price increases of SSB were associated with reductions in excess weight, especially for adolescent girls (46). However, whether these taxes achieve public health objectives depends, in part, on the extent to which beverage prices increase, known as tax pass-through. For example, given SSB profit margins are relatively high, restaurants or stores may be raising prices of both taxed and untaxed beverages (47, 48). In addition, to increase the sales of SSB, some restaurants employ strategies that may increase consumption, such as offering free refills (49). Second, updating the Nutrition Facts Label to communicate the amount of added sugars included in SSB (50) and introducing warning labels for SSB (51) could effectively dissuade consumers. Meanwhile, the updated or warning label may alter consumers' perceptions about the healthfulness of selected products (52). In addition, the school health team should provide children and adolescents with effective health awareness programs, including providing accurate information, such as appropriate sweet and soft drink amounts, restricting/reducing soft drink consumption in the schools, restricting selling soft drinks from the school premises, and offering a soft drink alternative by providing access to healthy drinks in the schools. In addition, effective social media reaching the younger population to make them aware of potentially harmful consequences of SSB should be applied (44). School health teams should also add the educational information to mental health content. Apart from this, clinicians or parents should not only focus on the diagnosis and treatment of obesity or high fat, and the deleterious effects including mental disorders caused by overweight or obesity need to be further paid attention, especially among children and adolescents.

The strengths of this study deserved to be mentioned. First, children and adolescents aged 7–17 years were included, with a wide age range. Moreover, since obesity might influence the mental disorders, we explored the potential effect of body composition on this issue, using the measure of dual-energy X-ray absorptiometry (DEXA), a previously validated measure in pediatric populations. Compared to BMI, body composition measures could better predict body size dissatisfaction in children and adolescents (53). Certain limitations should also be paid attention. First, the study population was selected in Beijing, and it could not be able to fully represent the Chinese children and adolescent population. Second, we could not rule out that certain types of foods, such as junk food (pastries, chocolates, candies, etc.), and some particular components, including saturated fat content, could also affect our findings. However, as for the information regarding energy intakes, trained project members interpreted all the questionnaires in detail, and appropriate guidance would be given by these project members as effectively as possible. The questionnaires would be rechecked by 3% within 1 week for the same participants. Therefore, the quality of the self-reported data regarding energy intake behaviors should be guaranteed. Third, a cross-sectional survey design could not generate casual relationships of drinking SSBs and depression or social anxiety. However, we also reported the potential “reverse causation” for the observed link between high SSB consumption and depression or social anxiety. In fact, it is not clear whether drinking SSBs or mental disorders occurred first, and a randomized controlled trial is desired to confirm our results. For example, children and adolescents with mental problems and who habitually consume SSB are randomly allocated to a 3-month no SSB provided diet intervention (Diet Group) or a habitual diet control group (Control Group), to investigate whether engaging in a diet intervention can reduce symptoms of mental problems.

## Conclusion

Children and adolescents in China were still exposed to high levels of depression symptoms and social anxiety, as well as high consumption of SSB. Higher consumption of SSB was positively associated with increased OR of mental health problems in children and adolescents, especially among those with high body fat or low FFM or muscle. This study added to evidence that avoiding soft drinks and beverages as much as possible and keeping a good body shape might be effective approaches to prevent mental disorders among children and adolescents. Thus, taxation of unhealthy foods, particularly sugar-sweetened food and beverage, and promotion of physical activity in keeping a healthy normal weight should be elements of policies to strengthen mental health systems in children and adolescents, which will also inversely support healthy behaviors, promote mental wellbeing, and prevent obesity-related potential metabolic diseases.

## Data Availability Statement

The raw data supporting the conclusions of this article will be made available by the authors, without undue reservation.

## Ethics Statement

The studies involving human participants were reviewed and approved by the Ethics Committee of Peking University (Number: IRB00001052 20024). Written informed consent to participate in this study was provided by students and their parents.

## Author Contributions

JL: conceptualization and writing—original draft. TC, MC, and YM: data curation. JL and MC: formal analysis. YD and JM: funding acquisition. JL, TM, DG, YL, and LC: methodology. YD and JM: project administration, resources, supervision, and validation. JL, XW, QM, and YZ: software. MC, YM, TM, DG, and YD: visualization. TC, YL, XW, QM, YZ, JM, and YD: writing—review and editing. All authors have read and agreed to the published version of the manuscript.

## Funding

This research was funded by the China Postdoctoral Science Foundation (BX20200019 and 2020M680266 to YD), the National Natural Science Foundation of China (82103865 to YD), and the Beijing Natural Science Foundation (7222244 to YD).

## Conflict of Interest

The authors declare that the research was conducted in the absence of any commercial or financial relationships that could be construed as a potential conflict of interest.

## Publisher's Note

All claims expressed in this article are solely those of the authors and do not necessarily represent those of their affiliated organizations, or those of the publisher, the editors and the reviewers. Any product that may be evaluated in this article, or claim that may be made by its manufacturer, is not guaranteed or endorsed by the publisher.
