# Supplementary material for: Sugar-Sweetened Beverages and Depressive and Social Anxiety Symptoms Among Children and Adolescents Aged 7–17 Years, Stratified by Body Composition
Source: Front Nutr. 2022 May 23;9:888671. doi: 10.3389/fnut.2022.888671 (PMC9168881; doi:10.3389/fnut.2022.888671)
Supplement: Supplementary file 1 [file Data_Sheet_1.pdf]

Supplementary Material

Supplementary Table 1. Multivariate odds ratios (OR) and 95% confidence intervals (CI) of depressive and social anxiety symptoms for SSB consumption of ≥1 servings /day.

SSB Consumption	None-depressive symptoms	Depressive symptoms	None-social anxiety	Social anxiety symptoms
Model 1				
**Total population**				
≥1 servings /day	1 (Reference)	**2.06 (1.31-3.26)**	1 (Reference)	1.17 (0.78-1.77)
**Boys (n=658)**				
≥1 servings /day	1 (Reference)	**3.26 (1.78-5.98)**	1 (Reference)	1.42 (0.82-2.46)
**Girls (n=653)**				
≥1 servings /day	1 (Reference)	1.20 (0.59-2.43)	1 (Reference)	0.93 (0.50-1.71)
Model 2				
**Total population**				
≥1 servings /day	1 (Reference)	**2.02 (1.23-3.31)**	1 (Reference)	1.11 (0.72-1.72)
**Boys (n=658)**				
≥1 servings /day	1 (Reference)	**2.86 (1.45-5.64)**	1 (Reference)	1.20 (0.65-2.20)
**Girls (n=653)**				
≥1 servings /day	1 (Reference)	1.37 (0.65-2.88)	1 (Reference)	1.00 (0.53-1.89)

Model 1: adjusted for age and sex.

Model 2: additionally adjusted for parental educational attainment, maternal smoking status, single-child status, BMI, incomes, fruit consumption, physical activity, screen time and the frequency of fried food consumption.

Bold values referred to $P < 0.05$.

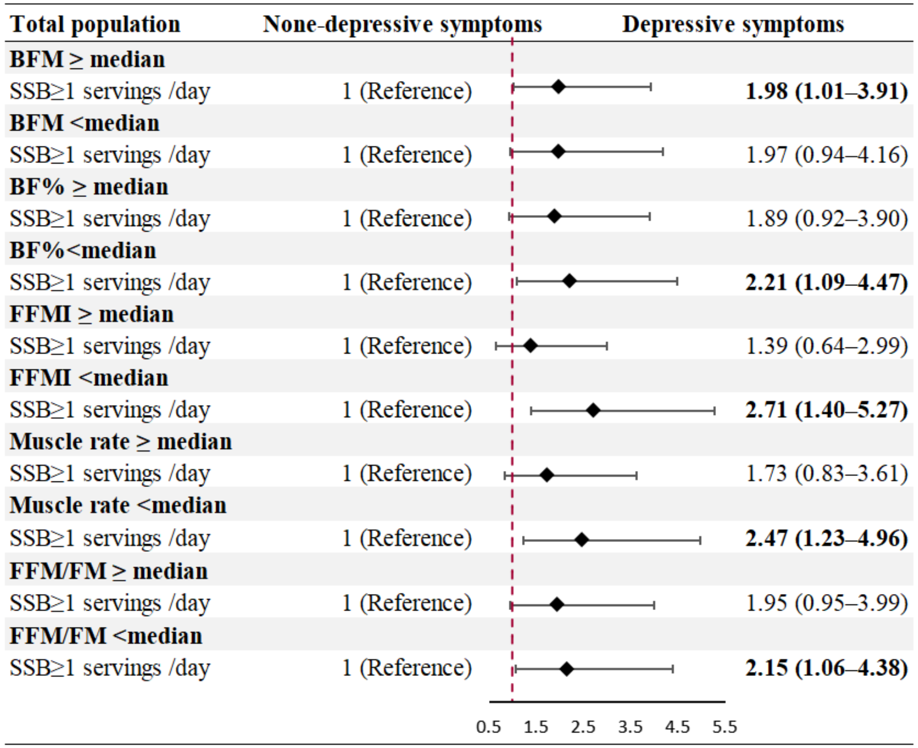
Total population
None-depressive symptoms
Depressive symptoms
BFM ≥ median
SSB≥1 servings /day
1 (Reference)
1.98 (1.01–3.91)
BFM <median
SSB≥1 servings /day
1 (Reference)
1.97 (0.94–4.16)
BF% ≥ median
SSB≥1 servings /day
1 (Reference)
1.89 (0.92–3.90)
BF%<median
SSB≥1 servings /day
1 (Reference)
2.21 (1.09–4.47)
FFMI ≥ median
SSB≥1 servings /day
1 (Reference)
1.39 (0.64–2.99)
FFMI <median
SSB≥1 servings /day
1 (Reference)
2.71 (1.40–5.27)
Muscle rate ≥ median
SSB≥1 servings /day
1 (Reference)
1.73 (0.83–3.61)
Muscle rate <median
SSB≥1 servings /day
1 (Reference)
2.47 (1.23–4.96)
FFM/FM ≥ median
SSB≥1 servings /day
1 (Reference)
1.95 (0.95–3.99)
FFM/FM <median
SSB≥1 servings /day
1 (Reference)
2.15 (1.06–4.38)
0.5
1.5
2.5
3.5
4.5
5.5

A

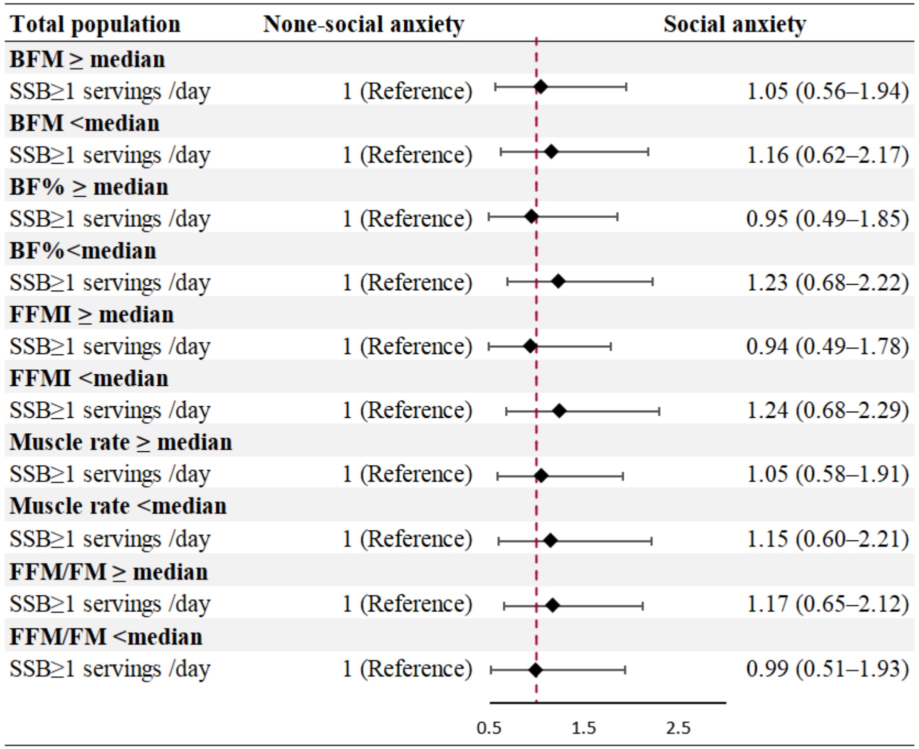
Total population
None-social anxiety
Social anxiety
BFM ≥ median
SSB≥1 servings /day
1 (Reference)
1.05 (0.56–1.94)
BFM <median
SSB≥1 servings /day
1 (Reference)
1.16 (0.62–2.17)
BF% ≥ median
SSB≥1 servings /day
1 (Reference)
0.95 (0.49–1.85)
BF%<median
SSB≥1 servings /day
1 (Reference)
1.23 (0.68–2.22)
FFMI ≥ median
SSB≥1 servings /day
1 (Reference)
0.94 (0.49–1.78)
FFMI <median
SSB≥1 servings /day
1 (Reference)
1.24 (0.68–2.29)
Muscle rate ≥ median
SSB≥1 servings /day
1 (Reference)
1.05 (0.58–1.91)
Muscle rate <median
SSB≥1 servings /day
1 (Reference)
1.15 (0.60–2.21)
FFM/FM ≥ median
SSB≥1 servings /day
1 (Reference)
1.17 (0.65–2.12)
FFM/FM <median
SSB≥1 servings /day
1 (Reference)
0.99 (0.51–1.93)
0.5
1.5
2.5

B

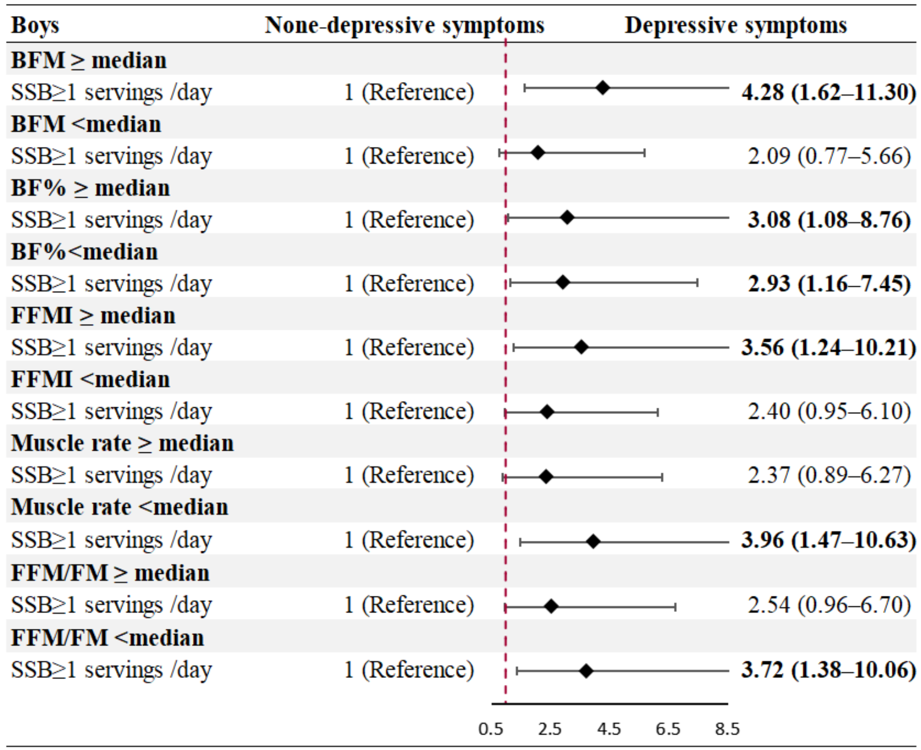
Boys
None-depressive symptoms
Depressive symptoms
BFM ≥ median
SSB≥1 servings /day
1 (Reference)
4.28 (1.62–11.30)
BFM <median
SSB≥1 servings /day
1 (Reference)
2.09 (0.77–5.66)
BF% ≥ median
SSB≥1 servings /day
1 (Reference)
3.08 (1.08–8.76)
BF%<median
SSB≥1 servings /day
1 (Reference)
2.93 (1.16–7.45)
FFMI ≥ median
SSB≥1 servings /day
1 (Reference)
3.56 (1.24–10.21)
FFMI <median
SSB≥1 servings /day
1 (Reference)
2.40 (0.95–6.10)
Muscle rate ≥ median
SSB≥1 servings /day
1 (Reference)
2.37 (0.89–6.27)
Muscle rate <median
SSB≥1 servings /day
1 (Reference)
3.96 (1.47–10.63)
FFM/FM ≥ median
SSB≥1 servings /day
1 (Reference)
2.54 (0.96–6.70)
FFM/FM <median
SSB≥1 servings /day
1 (Reference)
3.72 (1.38–10.06)
0.5
2.5
4.5
6.5
8.5

C

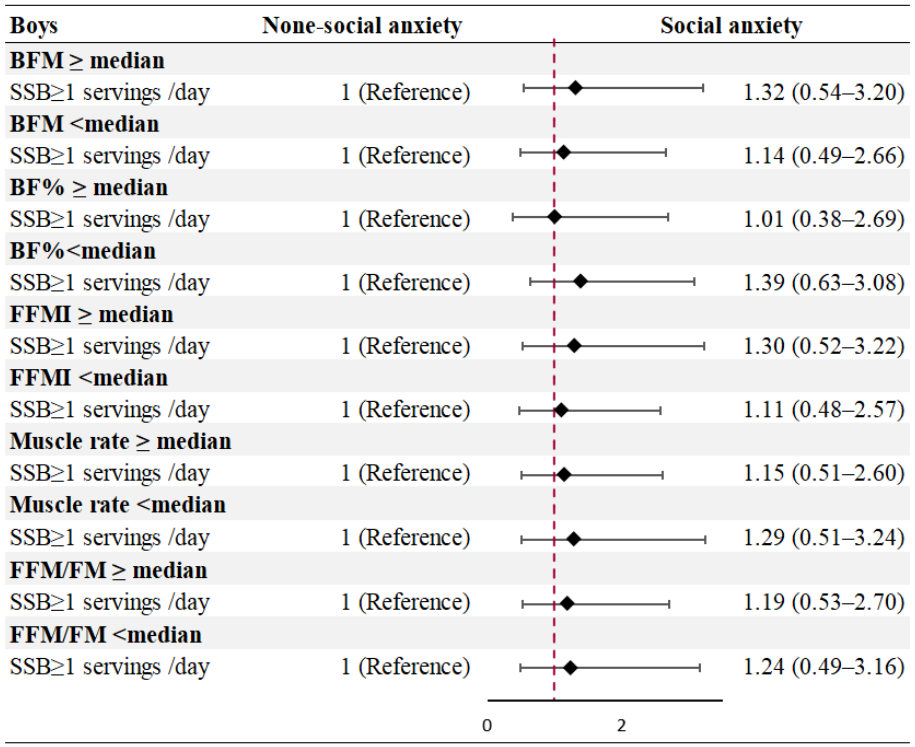
Boys
None-social anxiety
Social anxiety
BFM ≥ median
SSB≥1 servings /day
1 (Reference)
1.32 (0.54–3.20)
BFM <median
SSB≥1 servings /day
1 (Reference)
1.14 (0.49–2.66)
BF% ≥ median
SSB≥1 servings /day
1 (Reference)
1.01 (0.38–2.69)
BF%<median
SSB≥1 servings /day
1 (Reference)
1.39 (0.63–3.08)
FFMI ≥ median
SSB≥1 servings /day
1 (Reference)
1.30 (0.52–3.22)
FFMI <median
SSB≥1 servings /day
1 (Reference)
1.11 (0.48–2.57)
Muscle rate ≥ median
SSB≥1 servings /day
1 (Reference)
1.15 (0.51–2.60)
Muscle rate <median
SSB≥1 servings /day
1 (Reference)
1.29 (0.51–3.24)
FFM/FM ≥ median
SSB≥1 servings /day
1 (Reference)
1.19 (0.53–2.70)
FFM/FM <median
SSB≥1 servings /day
1 (Reference)
1.24 (0.49–3.16)
0
2

D

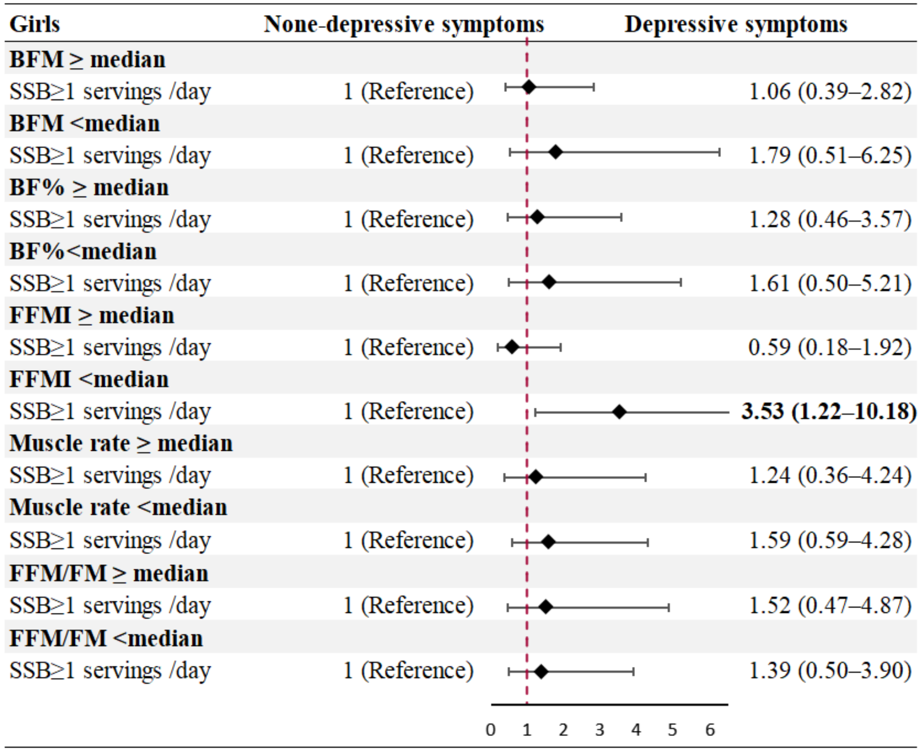

E

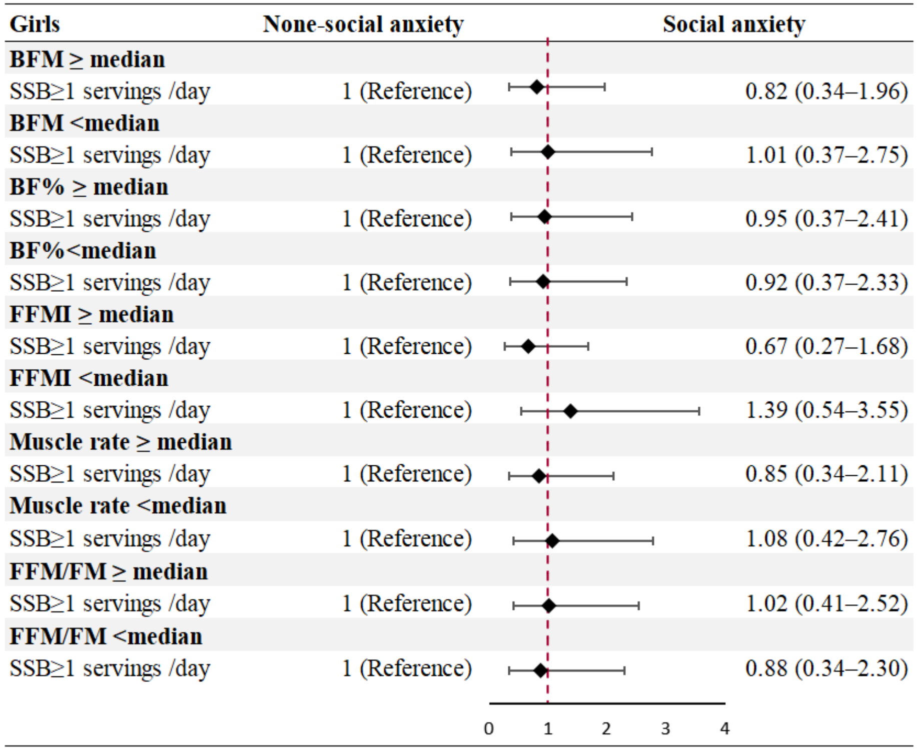

F

Supplementary Figure 1. Multivariate odds ratios (OR) and 95% confidence intervals (CI) of depressive (panel A, C, E) and social anxiety (panel B, D, F) symptoms for SSB consumption ≥1 servings /day among the total population and the each sex groups, stratified by body composition (Model was adjusted for age, sex, parental educational attainment, maternal smoking status, single-child status, BMI, incomes, fruit consumption, physical activity, screen time and the frequency of fried food consumption).
